# Exploring neuroanatomy and neuropsychology in digital financial decision-making: betrayal aversion and risk behavior

**DOI:** 10.1007/s11682-025-00967-1

**Published:** 2025-01-31

**Authors:** Santiago Carbó-Valverde, Raquel Martín-Ríos, Francisco Rodríguez-Fernández

**Affiliations:** 1 Faculty of Economics, University of Valencia, Valencia, Spain; 2 Faculty of Economics and Business, University of Granada, Campus Universitario de Cartuja, Granada, CP 18071 Spain; 3 Funcas, Madrid, Spain

**Keywords:** Betrayal aversion, Risk, Digital financial, Decision-making, Investment game

## Abstract

Detecting the factors associated with financial decision-making is an unresolved challenge when trying to predict digital financial behavior. This paper reports experimental results on both neuropsychological and neuronal correlates of risk-taking and betrayal aversion among 121 healthy participants (X_age_=21.7; SD = 2.8). A questionnaire on financial habits was administered, followed by neuropsychological tests and a magnetic resonance imaging session while participants viewed videos depicting both traditional and digital economic transactions. Participants also completed a computerized version of trust and risk games. Results reveal that Sensitivity to Punishment and Negative Urgency significantly predict risk behavior. High betrayal aversion was associated with greater sensitivity to punishment as well as lower volume in temporal areas. Cluster analysis identified two distinct psychological profiles based on betrayal aversion scores, highlighting differences in sensitivity to punishment, negative urgency, positive urgency, and trust game score. Furthermore, cortical thickness comparisons revealed differences between low and high-aversion groups, particularly in the temporal, parietal, and insular areas. White matter analysis indicated less integration in specific tracts among individuals with high betrayal aversion. These findings suggest the influence of neuropsychological factors on the adoption of risky financial behaviors and emotional response to betrayal.

**Supplementary Information:**

The online version contains supplementary material available at 10.1007/s11682-025-00967-1.

## Introduction

Financial decisions are essential for life. However, individuals tend to deviate from optimal financial behavior and frequently devote limited time on deliberating important financial decisions (Kuhnen & Knutson, 2005). Understanding the factors influencing financial decision-making in therefore paramount. Individual factors such as risk perception might have an impact on financial decision-making (Holt & Laury, 2002). According to Botterill and Mazur (2004), risk perception involves an individual’s subjective evaluation of the likelihood and potential consequences of uncertain events during decision-making. This perception often leads individuals to avoid or engage in activities or choices based on their perceived level of risk. Various internal factors, such as impulsivity or sensitivity to reinforcement or punishment, can influence the decision to engage in risky behavior (Botterill & Mazur, 2004). Impulsivity is conceptualized as a multidimensional construct that encompasses various personality traits leading to impulsive behaviors. These behaviors often occur without sufficient reflection, are oriented toward immediate goals, and typically neglect the consideration of potential consequences (Whiteside & Lynam, 2001) whereas sensitivity to punishment and reward reflects an individual’s propensity to respond to aversive and rewarding stimuli (Torrubia et al., 2001).

Neurofinancial studies have shed light on the neural correlates of risk-taking behaviour, highlighting the involvement of certain brain regions such as the nucleus accumbens (NAcc) and the insula. Activation of the NAcc has been associated with a greater propensity for risk-taking behavior, while insular activation is associated with a decrease in taking risks, which results in investments in safer assets (Kuhnen & Knutson, 2005). Insular activity also reflects a higher perception of risk which predisposes a person to exhibit a greater risk aversion (Smith et al., 2014). These results are consistent with other strands of research that find that both the anticipation of monetary rewards (Breiter et al., 2001; Izuma et al., 2008) are mediated by an activation of the nucleus accumbens (NAcc) of the ventral striatum.

Furthermore, the exposure to reward signals appears to modulate the nucleus accumbens triggering an anticipation of outcome, reward perception biases, and financial risk-taking (Knutson et al., 2008; Wu et al., 2012). This phenomenon would prove particularly important in contexts such as digital transactions which display a greater extent of continuous exposure to incidental reward cues.

On the other hand, research has indicated a greater aversion to risk-taking when outcomes depend on another player rather than on chance. Specifically, placing trust in another individual carries the inherent risk of potential betrayal (Bohnet & Zeckhauser, 2004; Bohnet et al., 2008). This phenomenon has attracted particular interest because economic interactions require trust. Betrayal aversion has been documented in several empirical studies using the Minimally Acceptable Probability (MAP) paradigm, where the acceptability thresholds were notably higher (i.e., more certainty was required to accept the risk) when outcomes were attributed to another individual rather than to chance (Humphrey & Mondorf, 2021).

Betrayal aversion may represent a separate construct different from risk aversion because betrayal is emotionally costly (Aimone, 2011). It is worth noting the study by Aimone et al. (2014) where differences in average blood oxygen level dependent (BOLD) activity were compared depending on whether transactions were made with a human versus computer counterpart. When participants decided to trust, an increased activity in the right anterior insular cortex and the medial anterior cingulate was observed. In addition, participants were divided into high aversion to betrayal and aversion to betrayal. The high aversion group reflected higher activity in the right anterior insular, right anterior frontal medial and right dorsolateral prefrontal cortex when playing with a human counterpart. The elevated insula activity would reflect the elevated negative state associated with betrayal aversion (Aimone et al., 2014).

Taken together, there is limited neural evidence about the mechanisms underlying the digital financial decision-making process (Carbó-Valverde et al., 2020). The aim of this study is to address this empirical gap by investigating questions with multiple neuropsychological, neural volumes, and financial decision-making measures. We aim to investigate both the neuropsychological factors and the neural correlates that underpin digital financial decision-making. We explore whether the degree of trust in traditional or digital financial transactions, impulsivity dimensions and sensitivity to punishment and reward scores can predict risk behavior. Secondly, we identify psychological traits and neuropsychological factors associated with the degree of betrayal aversion. Finally, we explore whether there are differences between cortical and subcortical volumes, depending on scores obtained in terms of the degree of the betrayal aversion. We would expect to find that digital transaction channels constitute good predictors of risk-taking behavior. As a second hypothesis, we would expect that the high betrayal aversion group will have a neuropsychological profile characterized by a pronounced sensitivity to reward. Finally, based on the role of the insula in emotional processing and decision-making, we propose the hypothesis that individuals with low aversion to betrayal will exhibit larger volumes in the left and right insula compared to those with high aversion to betrayal.

## Methods

### Participants

The sample was composed of one hundred and twenty-one healthy participants, with 53.72% being females (*n* = 65), whose ages ranged from 18 to 33 years old (X_age_= 21.7; SD = 2.8). They were recruited through media advertisements, and all of them completed an online survey on their financial habits. Participants were eligible if they had normal vision and above 18 years old. The exclusion criteria were: (1) any illness or mental disorders and (2) a concurrent dependence on substances (cocaine, heroin, alcohol, etc.).

### Measures

**The UPPS Impulsive Behavior Scale** (Whiteside & Lynam, 2001) was utilized, however we used the Spanish version (Cándido et al., 2012). The UPPS is a 20-item self-report questionnaire based on a five-factor: positive urgency, negative urgency, lack of premeditation, lack of perseverance, and sensation seeking. The 1-month test/re-test reliability of total UPPS scores in this sample was 0.87 (BPD *r* = 0.85; DD *r* = 0.89).

**The Sensitivity to Punishment and Sensitivity to Reward Questionnaire** (SPSRQ; Torrubia et al., 2001). The 48-item SPSRQ measures sensitivities to punishment and reward. Reliability of the SPSRQ was found to be satisfactory with alphas of 0.75 (SR) and 0.83 (Torrubia et al., 2001).

**Confidence in traditional vs. digital transactions.** In this study, risk perception specifically refers to the trust in financial transactions and how this trust varies between traditional and digital contexts. Consequently, we have employed an ad hoc measure to assess reliability. Participants were shown 18 videos with content reflecting traditional financial transactions (i.e. ATM, paying with cash or a credit card, etc.) and new digital transactions (i.e. PayPal, a mobile phone or a watch). After each video, participants rated their level of confidence with each type of transaction on a numerical scale from 1 to 4 (1 being “insecure/reliability” and 4 being “extremely secure/reliability”).

**Trust and risk games.** We implemented an adaptation of the trust (human counterpart) and risk (computer counterpart) games. The study involved a game setting where participants acted as investors, each round paired with a different, supposedly real participant (the trustee), under the pretense that these trustees were selected from previous participants to enhance realism and engagement. In every round, investors started with 12 points and could choose to send none, some, or all of these points to the trustee. Sent points were tripled, and trustees (either real or simulated in different game versions) could then return any portion of these increased points to the investor. The trust aspect was simulated by informing participants that the trustees had predetermined their responses to every possible point transfer. In a variation called the Risk Game, the role of the trustee was played by a computer algorithm, with the likelihood of point returns based on historical data from the Trust Game, removing the personal interaction element and focusing purely on risk assessment. Participants alternated between the Trust Game and Risk Game across 24 rounds without immediate feedback on returns, only learning outcomes after the first half and at the conclusion of all rounds. The Trust Game explored dynamics of trust and betrayal, as trustees could choose to reciprocate or betray the investor’s trust by keeping the transferred points, impacting the final points tally based on their decisions. The Risk Game, by contrast, stripped away the social interaction, with returns dependent on a programmed probability, illustrating differences in risk-taking behavior in a social versus non-social context (see Suplementary material).

### Procedure

An online survey was designed to ask the general population about their financial habits. All survey participants were offered the opportunity to participate in a second session in which some of the tests were administered. Participants underwent an MRI session while watching a series of videos on economic transactions (traditional and digital). Afterwards, they completed a computer version of the trust and risk game. The protocol described follows the structure described in a previous study (Carbó-Valverde et al., 2020).

### Imaging data acquisition and preprocessing

Neuroimaging data acquisition and preprocessing corresponds to the protocol described in our previous study (Carbó-Valverde et al., 2020). To acquire brain measurements, we used a 3 T Magnetom Tim Trio scanner supplied by Siemens Medical Solutions (Erlangen, Germany) equipped with a 32-channel receive-only head coil. For diffusion tension imaging (DTI) acquisition the parameters were: TR: 9,400 ms; TE: 88 ms; FOV: 256 mm; 72 slices; 2.0 × 2.0 × 2.0 voxel dimension; 30 volumes with diffusion weighting (b = 1,000 s/mm2) and one volume without diffusion weighting (b = 0 s/mm2). All images were inspected for correct processing. T1 image processing was conducted using the recon-all automated processing pipeline in Freesurfer (version 6.0). Cortical and subcortical volumes were automatically calculated based in the Destrieux atlas and the subcortical Freesurfer parcellation. Diffusion tensor images were preprocessed using FSL (Jenkinson et al., 2012) and included head motion and eddy-current induced artifacts correction, rotation of the gradient directions table and brain extraction. Fractional anisotropy (FA) and mean diffusion (MD) maps were calculated using the dtifit function. The automated AutoPtx (Groot et al., 2015) pipeline was used to run probabilistic tractography for some of the main system fibers in each individual. Complete details of the process are described elsewhere (Groot et al., 2015). All images were inspected for artifacts after acquisition. Outputs were also checked to discard outliers and incorrect processing. The software used to obtain the structural neuroimages data was the Statistical Parametric Mapping (SPM12) (Welcome Department of Cognitive Neurology https://www.fil.ion.ucl.ac.uk/spm/). Preprocessing with SPM12 includes realignment to the first image of the time series, co-registration to the structural image of each participant, unwarping, slice-timing correction, outlier detection, and normalization to an EPI template in the Montreal Neurobiological Institute (MNI) space.

### Statistical analyses

Firstly, a Stepwise Backward Multiple Linear Regression was conducted to identify the strongest predictors of risk-taking behavior (trust in traditional financial transactions, trust in digital transactions, impulsivity dimensions and reward and punishment sensitivity and demographic factors like Age, Gender, and Family Income. We dichotomized the betrayal aversion score into two categories high aversion (*n* = 66; 68% females) and low aversion (*n* = 53; 62% males) as a function of the overall mean. In addition, cluster analysis has been used to group different psychological traits according to the betrayal aversion cluster (reward and punishment sensitivity scale, impulsive personality traits, and risk and trust scores). Finally, we statistically compared cortical thickness values between low-aversion and high-aversion participants. A two-sample t-test was performed to compare whether there are differences in cortical and subcortical volumes between the groups. All statistical analyses were carried out using SPSS 25.0.

## Results

Frequency of sociodemographic variables was calculated as shown in Supplemental material. A multiple regression analysis was conducted to identify potential predictors of risky taking behavior. In Table 1, the regression analysis indicates that “Sensitivity to Punishment” (SP) and “Negative Urgency” (NU) are significant predictors of the risk game score. The model is statistically significant, F (4,113) = 3.63, *p* = 0.008, explaining 14.4% of the total variance. Specifically, Sensitivity to Punishment is positively related to the risk game score (B = 0.0287, t = 2.891, *p* = 0.005), and Negative Urgency also has a positive impact (B = 0.0604, t = 3.223, *p* = 0.002). In contrast, Lack of Premeditation (LPRE) shows a marginally significant positive effect (B = 0.0435, t = 1.726, *p* = 0.087), while Trust (Trust Game Score) has an inverse, but non-significant relationship with the risk game score (B = -0.0376, t = -1.661, *p* = 0.100). The Durbin-Watson statistic of 2.108 suggests no significant autocorrelation in the residuals.

**Table 1:** Summary of the multiple regression model

Predictor	Coefficient	Std. Error	t-value	*p*-value	95% Confidence Interval
Intercept	-0.7885	0.725	-1.087	0.279	[-2.227, 0.649]
SP	0.0287	0.010	2.891	**0.005**	[0.009, 0.048]
NU	0.0604	0.019	3.223	**0.002**	[0.023, 0.097]
LPRE	0.0435	0.025	1.726	0.087	[-0.006, 0.094]
Trust	-0.0376	0.023	-1.661	0.100	[-0.083, 0.007]

*Note*. *N* = 121; SP = Sensitivity to punishment; NU = Negative urgency; LPRE = Lack of premeditation; Trust = Trust Game Score

Regarding betrayal aversion, in the first step, we dichotomized the variable into two categories: high aversion (*n* = 66) and low aversion (*n* = 53) based on the total mean. Subsequently, the non-hierarchical K-means clustering method was used to segment the clusters to determine the psychological profiles according to the score obtained on betrayal aversion. The K-Means cluster analysis with two-cluster solution (iteration = 5) revealed that the low betrayal aversion group (Cluster 1: 44.6%, *n* = 53) was characterized by a mean score (M = 11) similar to the sample mean on Sensitivity to Reward, a lower mean (M = 7) than the overall mean (M = 11.01) on Sensitivity to Punishment, a higher mean (M = 12) than the overall mean (M = 10.87) in Negative Urgency, a higher mean than the sample mean in Lack of Premeditation (M = 8; M = 7.8), a lower mean score than the mean in Sensation Seeking (M = 9; M = 9.43) as well as a higher score in the Trust Game (M = 5.34) compared to the sample (M = 4.9) and the high aversion to betrayal group (M = 4.33). However, the low betrayal group was characterized by a similar score in Risk Play (M = 5.02) with respect to the total mean (5.1). Notably, Sensitivity to Punishment (*p* = 0.00), Negative Urgency (*p* = 0.019), Risk Game score (*p* = 0.00), and Trust Game score (*p* = 0.00) were statistically significant when compared to the total sample mean, indicating that these variables significantly distinguish the low betrayal aversion group from the overall sample.

On the other hand, the high betrayal aversion group (Cluster 2: 54.5%, *n* = 66) was characterized by a higher mean score (M = 12) than the total sample (M = 10.4) on Sensitivity to Reward, a higher mean score (M = 16) than the total mean (M = 11.01) in Sensitivity to Punishment, mean scores close to the mean in both Negative Urgency (M = 10) and Positive Urgency (M = 9), a higher mean score (M = 10) in Sensation Seeking with respect to the other cluster, and a higher score in Risk Play (M = 5.4) with respect to the total mean (5.1). In particular, Sensitivity to Reward (*p* = 0.038), Sensitivity to Punishment (*p* = 0.000), Negative Urgency (*p* = 0.007), Trust Game score (*p* = 0.000), and Risk Game score (*p* = 0.000) were statistically significant when compared to the total sample mean, indicating that these variables significantly differentiate the high betrayal aversion group from the overall sample. ANOVA revealed significant differences between clusters in the variable of Sensitivity to Punishment [F (1,115) = 226.687; *p* < 0.05], Negative Urgency [F (1,115) = 13.507; *p* < 0.05], Positive Urgency [F (1,115) = 4.209; *p* < 0.05] and the performance in the Trust Game [F (1,115) = 5.186; *p* < 0.05] (see Table 2).

**Table 2:** Mean scores for each cluster in the different tasks

Variables	Low	High	F	Sig.
SR	11	12	1.786	0.184
SP	7	16	226.68	0.000*
NU	12	10	13.50	0.000*
PU	10	9	4.209	0.042*
LPR	8	7	3.290	0.072
LPER	7	7	0.000	0.987
SS	9	10	0.147	0.702
Trust Game	5.34	4.33	5.186	0.025*
Risk Game	5.02	5.4	1.083	0.300

*Note*. *N* = 121; Low = Cluster 1; High = Cluster 2; SR = Sensitivity to reward; SP = Sensitivity to punishment; NU = Negative urgency; PU = Positive urgency; LPR = Lack of premeditation; LPER = Lack of perseverance; SS = Sensation seeking

Finally, we statically compared cortical thickness values between low-aversion and high-aversion participants. The brain volume analyses in our study focused on specific Regions of Interest (ROIs) identified based on previous research (Carbo-Valverde et al. 2020). Total intracranial volume was included as a covariate in these analyses to account for variability in participants’ head sizes (Barnes et al., 2010). We explored differences in both cortical and subcortical volumes (including frontal regions, striatum, insula, cingulate cortex, amygdala, and hippocampus) as a function of betrayal aversion through a two-sample t-test. Additionally, while the initial ROIs did not include the parietal and temporal lobes, our analyses revealed significant differences in these regions, which have now been incorporated into our discussion of the results. Analyses reflected that there are statistically significant differences between low-aversion and high-aversion groups in the left temporal volume [t (117) = 2.186, ρ < 0.05, d = 0.40]. In particular, the high-aversion group displayed the lowest mean volume reflected in this area. On the other hand, the low-aversion group was characterized by higher brain volumes in the left [t (117) = 2.467, ρ < 0.05, d = 0.45] and right parietal lobes [t (117) = 2.379, ρ < 0.05, d=. 43]. In addition, the low-aversion group reflected a higher mean volume in the left [t (117) = 2.155, ρ < 0.05, d = 0.39] and right insular volumes [t (117) = 2.557, ρ < 0.05, d = 0.47] when compared to the other group.

With regards to the quantitative characteristics derived from the diffusion images, we used fractional anisotropy (FA) to examine diffusion properties between different subjects (Basser & Jones, 2002) based on regions of interest. In our analysis, we considered relevant regions such as callosal fibers (forceps minor and major), fibers of the limbic system (cingulate gyrus and parahippocampal parts of the cingulum), association fibers (superior and inferior longitudinal fasciculus), and the corticospinal tract. To this point, we conducted a backward stepwise logistic regression to determine if there was a relationship between belonging to a particular group (high betrayal aversion vs. low betrayal aversion) and different fractional anisotropy measurements. In terms of CoxSnell’s R2, the overall model allowed us to estimate 11.6% in the variability of the criterion variable due to the relationship with the predictors. The Nagelkerke coefficient indicated that 15.5% of the variability is explained by the variables present in the model. Therefore, the fitted logistic model is adequate since χ^2^_*exp*_ = 14.624 (gl = 5) gives *p* = 0.00 > 0.05, and it correctly classifies 68.1% of the cases. Taking the high betrayal aversion group as a reference, the analysis reflected significant differences in the superior thalamic radiation (B= -32.490, *p* < 0.05) and inferior longitudinal fasciculus (B=-37.657, *p* < 0.05), indicating an inverse relationship with the criterion variable. On the other hand, posterior thalamic radiation FA (B = 35.487, *p* < 0.05) as well as the corticospinal tract FA (B = 34.878, *p* < 0.05) were statistically significant showing a direct relationship as seen in Table 3.

**Table 3:** Model coefficients

Predictor	B	SE	Wald	*p*	Odds ratio
Intercept	-1.21	6.05	0.040	0.842	0.298
FA_str	-32.490	15.867	4.193	0.041	1.67e-16
FA_ptr	35.487	13.801	6.611	0.010	2.582e + 15
FA_ilf	-37.657	14.490	6.753	0.009	2.63e-18
FA_cst	34.878	13.646	6.532	0.011	1.403e + 15

*Note*. Estimates represent the log odds of “High Betrayal Aversion = 2” vs. “Low Betrayal Aversion = 1”. FA_str = superior thalamic radiation; FA_ptr = posterior thalamic radiation; FA_ilf = inferior longitudinal fasciculus; FA_cst = corticospinal tract. *P < 0,05

## Discussion

The objective of the present study is to analyze risk-taking and betrayal aversion behaviors in financial digitization decisions. The results of the stepwise backward regression analysis reinforce our initial conclusions, while also providing additional nuances to the understanding of behavior in financial decision-making. In particular, although sensitivity to punishment (SP) proved to be a significant predictor, suggesting that individuals more sensitive to potential negative outcomes may adopt conservative behaviors in financial contexts, we also found a positive relationship between SP and scores on risk-taking games. This finding, which may appear contradictory, indicates that in simulated game contexts, individuals with high SP might engage in risk-taking behaviors as a coping or compensatory strategy. This result nuances the relationship between SP and decision-making, broadening the perspective on how aversion to punishment (or negative outcomes) influences different types of risk decisions. Negative Urgency (NU) was also found to be a significant predictor, highlighting the critical role that emotional impulsivity plays in shaping financial behavior. This finding suggests that individuals prone to impulsive actions under negative emotional states are more likely to engage in risky financial decisions. The significance of NU supports the hypothesis that emotional regulation is a vital component of decision-making, particularly in situations involving financial risk.

Interestingly, Lack of Premeditation (FPRE) and Trust Game Score (Trust) were identified as marginally significant predictors. While these variables did not exhibit the same level of influence as SP and NU, their inclusion in the final model suggests that a lack of foresight and the dynamics of interpersonal trust could also contribute to financial decision-making, albeit to a lesser extent. These findings introduce a layer of complexity to the original analysis, indicating that beyond impulsivity and sensitivity to punishment, other psychological traits may also play a role under certain conditions. On the other hand, variables such as Sensitivity to Reward (SR), Positive Urgency (PU), and demographic factors like Age, Gender, and Family Income were not retained in the final model. This suggests that their impact on financial behavior may be less significant in this context or may be mediated by other variables. This finding underscores the importance of focusing on the most impactful psychological traits, particularly SP and NU, when analyzing financial risk behavior.

While other variables such as Sensitivity to Reward (SR), Positive Urgency (PU), and demographic factors were not found to be significant in this analysis, their potential influence should not be entirely dismissed. Instead, these findings highlight the importance of context and the need for further research to explore the conditions under which these variables might become more relevant. Overall, the stepwise regression analysis not only confirms the robustness of the original findings but also expands the scope of the discussion to include additional factors that could influence financial decisions. These results suggest that while SP and NU are primary drivers of financial behavior, other factors such as premeditation and trust should not be entirely discounted.

Secondly, potential differences in neuropsychological traits and performance in the financial game were analyzed in relation to participants’ degree of betrayal aversion. The results revealed significant differences between the high and low betrayal aversion groups in sensitivity to punishment, negative urgency, positive urgency, and performance in the trust game. Specifically, the high betrayal aversion group obtained higher mean scores in sensitivity to punishment compared to the low betrayal aversion group. This neuropsychological parameter promotes behavioral avoidance and passive avoidance in situations related to the possibility of negative consequences or worry linked to harm from punishment or failure (Gray & McNaughton, 2008). Moreover, the low betrayal aversion group exhibited a greater willingness to take risks in the trust game and achieved significantly higher scores in terms of positive and negative urgency traits. Positive urgency is defined as the propensity to act impulsively in the presence of an intense positive affect, whereas negative urgency refers to the tendency to exhibit strong impulses when under the influence of negative affect conditions (Whiteside & Lynam, 2001). In line with Aimone et al. (2011), betrayal aversion arises from the desire to avoid adverse feelings (Aimone, 2011). These results are reminiscent of the Anticipatory Affect Model (Knutson et al., 2008; Wu et al., 2012) which postulates that incentive cues trigger an activation of the differentiable neural circuits. When the activation is associated with a positive affect (Nacc), this could promote an approach to a certain risky activity, whereas when has to do with to a negative affect (anterior insula) it may motivate an avoidance of risk. An alternative hypothesis emerging from neuroscience research assumes that risk behavior is associated with a dual-system model. This network would modulate the activity of a network underlying the reward-risk valuation associated with stimuli and, in parallel, be executed under another control network that mediates between the search for or avoidance of risky options (Steinberg, 2010). Based on the evidence above, our findings suggest that betrayal aversion may be associated with increased sensitivity to punishment and, consequently, with risk aversion. Meanwhile, low betrayal aversion is characterized by a willingness to take risks and impulsivity driven by emotional dysregulation, which conditions risk/benefit estimation. However, although several researchers argue that betrayal aversion can be understood as a risk-taking behaviour (Cook & Cooper, 2003; Bohnet et al., 2008), we consider that betrayal aversion may represent an independent but related dimension of the decision to engage or not in risk-taking behaviour.

Finally, the comparison of cortical thickness values between low-aversion and high-aversion participants revealed notable differences in brain volumes associated with betrayal aversion. In particular, the high-aversion group exhibited the lowest mean volume in the left temporal area, suggesting a potential association between heightened betrayal aversion and reduced volume in this area. The left temporal areas have been associated with performance in verbal memory and digital recall (Warrington & Weiskrantz, 1973). The reduced volume in this area may reflect alterations in cognitive functions related to language and memory, which could impact an individual’s ability to process and recall auditory information. Consequently, these alterations could also affect their ability to respond effectively, leading then to adopt a more cautious approach to social interactions. This cautiousness is likely driven by a heightened awareness of potential betrayal.

Conversely, the low-aversion group is characterized by higher brain volumes in both left and right parietal lobes, traditionally attributed to integrating sensorimotor information, and in the insular cortex, which plays a critical role in decision-making and perceptual awareness (Benarroch, 2019). In fact, the left parietal lobe is associated with mathematical performance. Specifically, the horizontal segment of the intraparietal sulcus is linked to an internal representation of quantities and magnitude processing (Serra-Grabulosa et al., 2010). Meanwhile, the parietal areas are directly involved in the execution of working memory-demanding tasks (Kandel et al., 2000) whose performance is related to more sophisticated arithmetic procedures for further processing of problem solving (Passolunghi & Pazzaglia, 2004).

Furthermore, the low-aversion group demonstrated increased volumes in the left and right insular regions compared to the high-aversion group. The insula is known to play a crucial role in processing emotions and decision-making, particularly in situations involving risk and uncertainty (Benarroch, 2019). Considering the differences observed in the insula between the groups, it suggests that the phenomenon of betrayal aversion promotes a distinct pattern in emotional processing as a response to the perceived threat of betrayal.

Therefore, the observed differences in insular volumes between the low- and high-aversion groups may reflect variations in emotional processing and risk assessment strategies. Indeed, these results align with the previously described neuropsychological profile, which suggested that the group with low betrayal aversion modulated their behavior based on emotional signals, such as positive and negative urgency. Specifically, a group exhibiting high urgency scores, indicative of more hasty and impulsive behavior in emotionally charged situations, may display a more reactive approach to decision-making. This could facilitate a greater influence of emotional signals on their decisions.

Regarding the analysis of white matter anisotropy, the high-aversion group showed less integration in both superior thalamic radiation and the inferior longitudinal fasciculus (ILF) indicating a potential decrease in white matter integrity in these regions. The association fibers ILF connect the anterior pole of the temporal lobe with the cortical end of the occipital lobe. This fasciculus has been associated with object and face recognition as well as empathy and emotional contagion (Comes-Fayos et al., 2018). In addition, they also showed greater integration in both posterior thalamic radiation and the corticospinal tract, whose efferent projection fibers are part of the internal capsule and are linked with fine psychomotor performance and accuracy (Snell, 2007). Even if the data may suggest some similarity between the phenomenon of betrayal aversion and risk-taking behaviors, it is important to note that we are dealing with a phenomenon that exhibits certain peculiarities: an emotional nuance and moral connotations. On the other hand, a limitation of the study is the lack of specific analysis on cluster homogeneity regarding sociodemographic variables, such as age and gender, which could influence the interpretation of the results.

In conclusion, the results confirm the importance of psychological traits such as Sensitivity to Punishment (SP) and Negative Urgency (NU) in financial decision-making, particularly in risk-taking and impulsive decisions under emotional distress. These findings emphasize the role of SP and NU in shaping individuals’ approaches to financial risk and suggest that other factors, such as Lack of Premeditation (LPRE) and Trust Game Score (Trust), although less influential, may also be relevant in specific contexts. This broadens our understanding of the factors that influence financial decision-making, revealing that financial behavior may be shaped by a more diverse set of psychological and interpersonal factors. Ultimately, this comprehensive analytical framework establishes a foundation for future research to further explore how these psychological traits interact in various financial contexts.

## Electronic supplementary material

Below is the link to the electronic supplementary material.

Supplementary Material 1

## Data Availability

The datasets generated and analyzed during this study are available from the corresponding author on reasonable request.
