# Supplementary material for: Exploring neuroanatomy and neuropsychology in digital financial decision-making: betrayal aversion and risk behavior
Source: Brain Imaging Behav. 2025 Jan 31;19(2):357–64. doi: 10.1007/s11682-025-00967-1 (PMC11978724; doi:10.1007/s11682-025-00967-1)
Supplement: Supplementary file 1 — Supplementary Material 1 [file 11682_2025_967_MOESM1_ESM.docx]

**Trust game**

Participants played the role of “investor”. To increase the credibility of the game, participants were told that their possible “trustee” was another randomly selected participant in the experiment. In each trial, participants received an initial endowment of 12 points, of which participants could give either 0, 4, 8 or 12 points to the trustee. Their trustee would receive their endowment multiplied by three. On the other hand, the trustee had the option to return any amount between zero and their total available amount to the investor (which would depend on the participant’s prior decision). The amount returned by participant B will not be multiplied by 3. Participants made their selections by pressing one of the four buttons we provided, indicating the number of points to transfer. Subsequently, a new screen appeared for eight seconds informing the participants that the administrators were in the process of making their decision. Finally, each trial ended with a fixation crossover screen with a variable duration of 10-12 seconds.

**Risk game**

Participants were faced with the same choices as in the trust game. However, in this game the “investors” were paired with a random computer mechanism in the role of “trustee”, rather than with another participant in the experiment. Participants had to decide what money to invest in a lottery. The amounts they might allocated to the lottery were 0 points, 4 points, 8 points or 12 points. The points they would decide to invest in the lottery are multiplied by 3 and would add up to 12 points. Therefore, participants knew that a computer would determine whether or not to return points based on the probability distribution generated by the trustees' decisions in the trust game. Thus, for each quantity returned we assigned a probability. In this sense, if the investor invested 8 points in the lottery in this experiment, he could receive back 0 points with a probability of 0.4, 8 points with a probability of 0.3, 16 points with a probability of 0.2 and 20 points with a probability of 0.1. After the first 12 rounds of both risk and trust games, participants received information on the number of times their partner (person or computer) had returned their points. At the end of the experiment, participants were informed about the total number of points they scored in each trial. The central difference between trust and risk games is that while in the risk game the investor's potential risk depends on a random mechanism, while in the trust game it relies on the uncertainty about a social interaction with a real person in the role of trustee.

Experimental evidence shows that individuals have an aversion to being betrayed (Bohnet et al., 2008). Therefore, in our experiment, investors displaying a lower risk-taking tendency in the trust game as opposed to the risk game, indicates what we interpret as “betrayal aversion”. Based on this, the amount of points transferred was collected on a trial-by-trial basis. The average amounts sent in the trust and risk games were calculated, respectively. In addition, to further examine the phenomenon of betrayal aversion, we compared the points transferred in each game and then calculated a betrayal aversion score by subtracting the amount transferred in the risk game from the amount transferred in the trust game.

**Instructions for experimental subjects**

In this section we include the experimental instructions (translated from the original ones in Spanish) for the person experiment (trust game) and the instructions for the lottery experiment (risk game).

INSTRUCTIONS

Welcome and thank you for participating in this study. In this document you will find the instructions for the experiments you will perform next. You should be aware that there are no right or wrong answers. Your identity will remain anonymous at all times and only the answers you provide will be used.

The purpose of these experiments is to study how individuals make decisions in certain contexts. The instructions are simple and it is essential that you understand them properly. If you have any questions, do not hesitate to ask the person in charge. Depending on the decisions you make and the decisions made by the different people you will be paired with, you will earn more or less money.

Instructions for the person experiment

In this game there are two stages and two types of participants: A and B.

You will be participant A and the person you are paired with will be participant B. Both participants will receive an amount of 12 points.

Stage 1.

- As participant A you have to decide: you have to decide how much of the 12 points to send to participant B and how much to keep. The amounts you can send are 0 points, 4 points, 8 points or 12 points.
- All points sent to Participant B will be multiplied by 3 before they receive them. If you send 0 points to participant B you will receive 0 points, if you send 4 points to participant B you will receive 12 points, if you send 8 points to participant B you will receive 24 points and if you send 12 points to participant B you will receive 36 points.

Stage 2.

- Participant B must decide how many points to keep and how many to send to Participant A (from your initial 12 points plus the amount sent by you, multiplied by 3 in stage 1). The amount returned by participant B will not be multiplied by 3.
- The person you are paired with has already decided as a B participant. However, this person did not know the amount of points that you, as participant A, would send to him/her. When you now decide in step 1 which amount to send to participant B, we will associate this amount with the decision B made for this sending option.

Both your and participant B's earnings in this experiment therefore depend on the decisions of both participants. As participant A you earn the amount of points initially kept plus the amount sent by participant B. Participant B earns the amount of points he/she keeps.

TOTAL EARNINGS

This experiment consists of 12 rounds and in each round, you will be paired with a different person. Both you and the participants with role B will be paid according to the following conversion rate: €1 = 25 points.

Lottery experiment instructions

In this game there is one stage and only one player A. As participant A you will receive an initial amount of 12 points.

- As participant A you must decide how much of this money to allocate to a lottery and how much to keep. The amounts you can allocate to the lottery are 0 points, 4 points, 8 points or 12 points.
- Any points you spend on the lottery are multiplied by 3 and 12 points are added. If you allocate 0 points they will be transformed into 12 points, if you allocate 4 points they will be transformed into 24 points, if you allocate 8 points they will be transformed into 36 points and if you allocate 12 points they will be transformed into 48 points.
- Therefore, depending on the amount you decide to allocate to the lottery, you can receive back from 0 points to the maximum amount in each case. In other words, if you allocate 0 points to the lottery, you can receive back from 0 points to 12 points. If you allocate 4 points to the lottery, you can receive back from 0 to 24 points. If you allocate 8 points to the lottery you can receive back from 0 points up to 36 points. And if you allocate 12 points to the lottery you can receive back from 0 points to 48 points.

The amount of money you receive back will depend on the decisions made by all participants B in the previous experiment. We have grouped the amounts returned by all participants B from the previous experiment for each amount sent by participants A (0 points, 4 points, 8 points or 12 points). For each amount returned we have assigned a probability. The probability that in this experiment you will get one of the amounts depends on the percentage that participant B chose to return on a case-by-case basis (0 points, 4 points, 8 points or 12 points) in the previous experiment.

For example, suppose that in the previous experiment, when participant A chose to send back 8 points, the amounts that participant B sent back were as follows: 40% sent back 0 points, 30% sent back 8 points, 20% sent back 16 points and 10% sent back 20 points. So, if in this experiment you decide to send 8 points to the lottery, you can receive back 0 points with a probability of 0.4, 8 points with a probability of 0.3, 16 points with a probability of 0.2 and 20 points with a probability of 0.1.

TOTAL EARNINGS

As participant A you earn the amount of points you initially kept plus the amount you win in the lottery. This experiment consists of 12 rounds. You will be paid according to the following conversion rate: 1€=25 points.

Thank you very much for your collaboration in this Project.

**Table 1. Means and standard deviations.**

| Variables | Mean | SD | Minimum | Maximum |
| --- | --- | --- | --- | --- |
| Age | 21.73 | 2.80 | 18 | 33 |
| Sensitivity to reward | 10.40 | 5.50 | 0 | 23 |
| Sensitivity to punishment | 11.01 | 4.10 | 1 | 19 |
| Negative urgency | 10.87 | 2.91 | 5 | 16 |
| Positive urgency | 9.75 | 2.07 | 5 | 14 |
| Lack of premeditation | 7.86 | 2.51 | 4 | 16 |
| Lack of perseverance | 7.47 | 2.60 | 4 | 16 |
| Sensation Seeking | 9.43 | 2.42 | 4 | 15 |
| Trust Game | 4.93 | 2.37 | .00 | 12 |
| Risk Game | 5.18 | 1.95 | .67 | 11.67 |
| Betrayal aversion | .25 | 2.59 | -8 | 7.67 |

**Note.** N=121

.
